# Electrical Signaling, Photosynthesis and Systemic Acquired Acclimation

**DOI:** 10.3389/fphys.2017.00684

**Published:** 2017-09-14

**Authors:** Magdalena Szechyńska-Hebda, Maria Lewandowska, Stanisław Karpiński

**Affiliations:** ^1^Department of Plant Genetics, Breeding and Biotechnology, Warsaw University of Life Sciences Warsaw, Poland; ^2^The Franciszek Górski Institute of Plant Physiology, Polish Academy of Sciences Krakow, Poland

**Keywords:** electrical signal, ion channel activity, plasma membrane, photosynthesis, PsbS overexpression and *npq-4*

## Abstract

Electrical signaling in higher plants is required for the appropriate intracellular and intercellular communication, stress responses, growth and development. In this review, we have focus on recent findings regarding the electrical signaling, as a major regulator of the systemic acquired acclimation (SAA) and the systemic acquired resistance (SAR). The electric signaling on its own cannot confer the required specificity of information to trigger SAA and SAR, therefore, we have also discussed a number of other mechanisms and signaling systems that can operate in combination with electric signaling. We have emphasized the interrelation between ionic mechanism of electrical activity and regulation of photosynthesis, which is intrinsic to a proper induction of SAA and SAR. In a special way, we have summarized the role of non-photochemical quenching and its regulator PsbS. Further, redox status of the cell, calcium and hydraulic waves, hormonal circuits and stomatal aperture regulation have been considered as components of the signaling. Finally, a model of light-dependent mechanisms of electrical signaling propagation has been presented together with the systemic regulation of light-responsive genes encoding both, ion channels and proteins involved in regulation of their activity. Due to space limitations, we have not addressed many other important aspects of hormonal and ROS signaling, which were presented in a number of recent excellent reviews.

## Introduction

One of the most critical functions of each organism is a selective sensing of the environment. Ordered flow of electrical currents between cells and organs allows a given organism for universal, rapid, and efficient communication of the external changes. The steady state of plasma membrane electrical potential defines the electric field of each cell. However, external factors induce rapid changes in the membrane potential, and these changes can be transduced in the form of waves: (1) the movement of ions across of plasma membrane and organelle membranes is a driving mechanism for wavy changes of the electric potential, which propagate along the membrane of one cell or organelle, and in turn, determine intracellular electrical activity of the cell and adjust its local metabolism; (2) the short-distance intercellular electrical signaling to maintain specific behavior of the group of the cells; and (3) the long-distance intercellular electrical signal from the site of stimulus perception to distal organs, where it triggers plant-wide responses.

Despite specific differences, the network of electrical signaling is present at almost each level of complexity, from unicellular bacteria and fungi to multi-cellular organisms like plants and animals. In unicellular organisms, cell-to-cell electrical signaling plays a key role in the reproduction and coordination of colony behavior. For example, bacteria *Bacillus subtilis* generates electrical signals mediated by potassium ion channels to direct motility in a biofilm of their own community, to stop reproducing bacteria on colony periphery, and to leave core cells with a sufficient nutrient supply (Humphries et al., 2017). A polarization and dynamic coordination of the electrical signals underlies also the ability of plant cell groups to proliferation, proper morphogenesis, regeneration and orientation (Filek et al., 2002; Yan et al., 2009; Nakajima et al., 2015). Similarly, the bioelectric network of each cell and the bioelectric gradients serve as a kind of pattern memory of animal tissues and organs (Durant et al., 2017). The environmental signals, physical (e.g., light, temperature, humidity, electric fields, wounding), chemical (e.g., nutrients and various substances), and biological (e.g., symbiosis, pathogenesis), can alter local and systemic electrical responses and modify cell division and growth. However, once the connectivity patterns of electrical signaling are disrupted, organisms can no longer follow appropriate morphogenetic and functional pathways (Szechyńska-Hebda et al., 2010; Karpiński et al., 2013; Nakajima et al., 2015).

Probably the most spectacular system involving electrical signaling is the organism-to-organism signaling. Among unicellular bacteria, electrical communication enables cross-species interactions. *Pseudomonas aeruginosa* cells become attracted to the electrical signal released by the *B. subtilis* biofilm (Humphries et al., 2017). In the plant kingdom, the role of electrical signals in organism-to-organism interactions is still highly speculative and largely phenomenological, but there are several pioneering examples of how plant creates and responds to electrical fields. Flowers exhibit differences in the pattern of the electric field, which can be discriminated by bumblebees. When the bumblebee lands on the flower, the electric field changes within seconds and this facilitates rapid and dynamic signaling between flowers and their pollinators (Clarke et al., 2013). *Arabidopsis thaliana* respond to biotic stress agents: *Spodoptera littoralis, Myzus persicae, Pseudomonas syringae* with plasma membrane depolarization and it was correlated to specific regulation of the wide range of defense genes (Bricchi et al., 2012). Similarly, transition zone of the roots is an area with unusually high levels of electrical activity (Baluška, 2010; Baluška and Mancuso, 2013), and it makes the root apex zone an attractive target of pathogenic and symbiotic organisms (Brenner et al., 2006). There is also the possibility that electric field generated by each growing root might allow electrical signaling among roots of the same or another plant (Schenk and Seabloom, 2010; Garzon and Keijzer, 2011). However, the most extremal example among multicellular organisms, is the usage of electric organs by fish in murky environment to navigate, recognize the species and sex, and as a shocking defense (Gallant et al., 2014). The electric field generated for predatory purposes is up to 500 V or higher.

## Systemic propagation of electrical signals in plants

All plants generate long-distance electrical signals, and these signals serve for communication and integration of responses in different tissues and organs. The most extensively studied are electrical signals in lower plants, e.g., *Characeae*or and in higher “sensitive” plants such as *Mimosa pudica* or *Dionaea muscipula*. However, in “ordinary” plants, a variety of electrical phenomena have also been described. Various treatments can trigger a specific pattern of changes in the plasma membrane electrical potential of *Arabidopsis* (Szechyńska-Hebda et al., 2010), *Vicia faba* (Dziubińska et al., 2003a; Zimmermann et al., 2009), *Triticum aestivum* (Dziubińska et al., 2003b), *Zea mays* (Grams et al., 2007), as well as in tree species like *Salix* (Fromm and Spanswick, 1993), *Populus* (Lautner et al., 2005), and *Persea* (Oyarce and Gurovich, 2010).

The electrical signals induced by external stimulus differ in their spatial and temporal pattern, in mechanism of the activation, and evoked responses. First, local electrical potential (LEP) is a sub-threshold response induced by environmental factors (e.g., light, cold, water status changes, phytohormones, pathogen infection). LEP is not transferred to other parts of a plant, but has an impact on the local physiological status of the cell (Yan et al., 2009; Roux et al., 2014). Second, action potential (AP) is a fast “all-or-nothing” signal, locally generated in the cell after treatment with different stimuli (light/darkness, electrical stimulation, cold, mechanical stimulus), provided that stimulus reaches a certain threshold. Mechanism of AP propagation involves membrane depolarization and subsequent repolarization, depended on passive Ca^2+^, Cl^−^, and K^+^ ion fluxes. Activation of potential-dependent Ca^2+^ channels is probably the first stage of AP generation. AP propagates to distant organs without loss of amplitude and triggers a systemic response associated with transient changes in gas exchange, carbon assimilation process, respiration rate, reduction in phloem transport, and expression of specific genes. The information within the transmitted signal may be encoded by the shape of a single AP determined by the relative contribution of the ionic conductance; the AP amplitude (in range of 10–80 mV); the frequency of multiple AP signals and refractory period, i.e., the period following the AP when the cell cannot be stimulated (Fromm and Lautner, 2007; Hedrich, 2012; Kupisz et al., 2017). Third, variation potential (VP), also called electropotential wave is a slow signal evoked by local damaging stimulations (wounding, heating, burning). VP signals also consist of transient changes in membrane potential, but they are irregular in shape and longer in duration (the repolarizations is delayed). Amplitude of VP depends on type and intensity of the damaging stimulus. The VP ionic mechanism differs from that underlying APs; it mainly depends on transient P-type H^+^ ATPase inactivation in the plasma membrane. However, passive Ca^2+^ influx and Cl^−^ channel activation were also considered to be involved in VP generation. Probably, activation of ligand-dependent (chemical mechanism) or mechano-sensitive (hydraulic mechanism) calcium channels allows Ca^2+^ influx, which in turn triggers both H^+^ ATPase inactivation and anion channel activation. Therefore, VP propagation parameters might be under control of a hydraulic waves or/and chemical agent (wound substances). When transmitted systemically to other organs of the plant, VP influences the quantum yield of electron transport through photosystem II, the net CO_2_ uptake rate, respiration, jasmonic acid concentration, ethylene emission, gene expression and protein synthesis. Taken together, VPs seems to carry more information than the APs (Stankovic et al., 1997; Vodeneev et al., 2015, 2017; Gilroy et al., 2016). Fourth, system potential (SP) is a self-propagated systemic signal mediated by the apoplastic ions and the plasma membrane H^+^-ATPase. The initial polarity of these voltage-dependent signal is hyperpolarization. In contrast to action or variation potentials, all of the ions (Ca^2+^, K^+^, H^+^, and Cl^−^) are involved in SP propagation, after the voltage change begins. SP does not obey the all-or-none rule but depends on the intensity and nature of the original stimulus (Zimmermann et al., 2009).

Dependently on the type of stimulus and plant species, electrical signals are transferred to distant tissues and organs with different speed. AP velocity has been estimated in range of 4–8 to 70 mm s^−1^ for green algae and higher plants, and even up to 400 mm s^−1^ in woody plants (Volkov, 2000, 2006; Fromm and Lautner, 2007). VP is always slower than AP; the flaming or wounding of higher plants evoke VP signal, which moves at a speed of 0.5–5 mm s^−1^ (Fromm and Lautner, 2007; Chen et al., 2016). The SP propagates at 0.8 to 1.7 mm s^−1^ from leaves that had been injured by cutting (Zimmermann et al., 2009). In *Arabidopsis thaliana* different types of the electrical signals can be generated by various stimuli. The repetitive APs together with VP signal can be induced in the leaf by wounding and KCl treatment. After this stimulus, APs propagated in a bidirectional manner (but mostly from the wound to the petiole) with the velocity approx. 0.5–4 mm s^−1^, whereas VP was recorded as an unidirectional slow propagation wave with the velocity 0.22–0.26 mm s^−1^ (Favre et al., 2001). Similarly, AP-like depolarization wave was recorded together with the slow depolarization wave corresponding to a VP in the sieve elements, when *A. thaliana* leaves were wounded. The electrical signal propagation had a velocity of c.a. 0.3–2.5 mm s^−1^ (Salvador-Recatal et al., 2014). Touch of *A. thaliana* leaves induced only APs, that moved at a speed of 1.3 mm s^−1^ (Agosti, 2014). Voltage-elicited APs for *A. thaliana* ecotype Columbia propagated from the stimulus area via the petiole to the central axis of the rosette with velocity ranging from 0.8 to 1.9 mm s^−1^, whereas for the Wassilewskija ecotype the propagation speed was 0.76–0.17 mm s^−1^. Moreover, the results for both ecotypes differed markedly in the general occurrence of APs; 91% plants ecotype Col and only 45% plants ecotype *Ws* responded to electrical stimulation with generation of the APs (Favre and Agosti, 2007). Several seconds of excess light illumination have induced a systemic electrical signal corresponding to VP or SP. This signal has propagated between two different leaves with the speed of 2 mm s^−1^. Switching light off has triggered signals with the speed ~3 mm s^−1^ (Szechyńska-Hebda et al., 2010).

**Figure 1:**
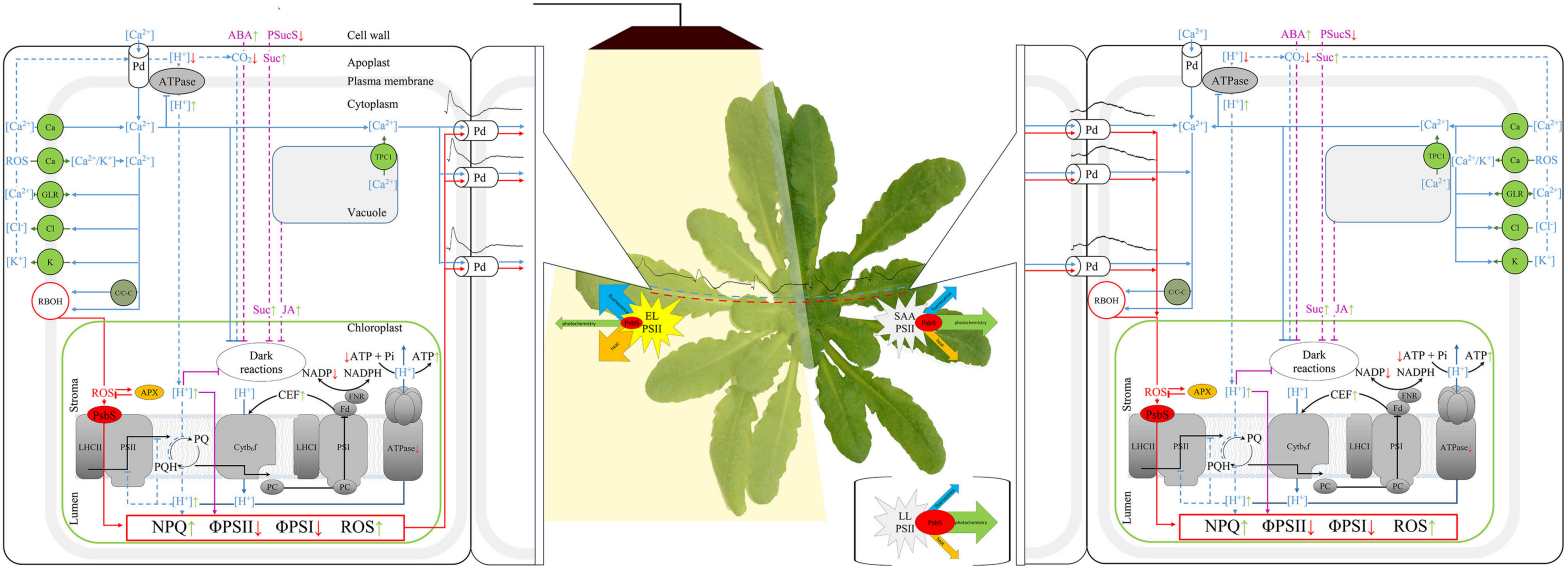
Scheme of the signaling pathways triggered by electrical signals, that are possibly involved in long-term adjustment of photosynthesis, SAA and SAR. The blue solid lines indicate the induction of the calcium wave; blue dotted lines—fast pathways influencing photosynthesis; purple lines - the pathways for long-term inactivation of photosynthesis; red lines—ROS- and NPQ-dependent pathways, sinus-like wave—the electric signals, black solid lines—electron flow. Abbreviations in the alphabetical order: ABA, abscisic acid; APX, ascorbate peroxidase; arrows, increase (green) and decrease (red) of a process; ATPase, ATP synthase; C/C-C, Ca^2+^- dependent protein kinases (CPK/CBL-CIPKs); Ca, potential-dependent, mechano-sensitive and (or) ligand-dependent Ca^2+^ channels; CEF, cyclic electron flow; Cl, Ca^2+^- dependent Cl^−^ channels; Cyt b_6_f, cytochrome b_6_f complex; EL, excess light; FD, ferredoxin; FNR, ferredoxin-NADP^+^ reductase; GLR, Glue receptor-like channels; JA, jasmonic acid; K, potential-dependent K^+^ channels; LHCII and LHCI, Light-harvesting complex II and I; LL, low light; NPQ, non-photochemical quenching; Pd, plasmodesma; PQ and PQH_2_, oxidized and reduced plastoquinone; PsbS, Photosystem II Subunit S; PSII and PSI, photosystems II and I; PSucS, phloem sucrose symporter; RBOH, Respiratory oxidase homolog; SAA, systemic acquired acclimation; Suc, sucrose; TPC1, vacuolar TWO PORE CHANNEL1 calcium channels; ΦPSI and ΦPSII, the photochemical quantum yield of PSI and PSII.

The electrical signal transmission in the living system plays the central functional role as it elicits systemic responses in an unaffected tissue to protect or defend the whole plant from a second occurrence of that same or tightly associated stress. The fact that similar electrical signals appear in response to many different stimuli suggests that stimulus-specific signals are encoded within the spatial and temporal dynamics of these waves. However, they may act as initial, general priming signals, preparing the plant to respond in a more selective way to subsequent, stimulus-specific signals (Choi et al., 2017). In this case, a number of specific mechanisms and signaling systems have been proposed to operate in combination with electric signaling, e.g., calcium and ROS waves; rapid changes in the xylem pressure; the level of photosynthetic products; hormonal circuits; peptide, protein and RNA signals (Shabala et al., 2016). Operating at different timescales, they encode information about the specific nature of a particular stimulus. The [Ca^2+^]_cyt_ signals are very rapid, approx. 400 μm s^−1^. The lower steady state Ca^2+^ concentration (~100 nM) in comparison to extracellular space and endoplasmic reticulum interior, a large number of Ca^2+^ ion channels in cellular membranes, and a broad range of Ca^2+^ sensor proteins, all of these factors allow the calcium signal to convey information about the nature and amplitude of external stimuli. Similarly, ROS propagation is relatively fast, approx. 8 cm min^−1^ (Miller et al., 2009). AP can induce the formation of free radicals, and ROS are known to be regulators of a broad range of cation and anion channels, thus a cross-talk between electric and ROS signals is quite probable (Shabala et al., 2016). Plants are able to induce very different and specific cell responses by using a small number of ROS molecules. ROS can act directly as “signaling molecules” or indirectly as “secondary products.” Nevertheless, additional aspects of the ROS adjustment during sensing and signaling mechanisms need to be considered: molecule type, its concentration and cellular localization, or a combination of all of these; modulation of ROS signaling by antioxidative enzymatic system, consisting of specific components in different cellular compartments (Szechyńska-Hebda and Karpiński, 2013). Further, the electrical signals can be accompanied by rapid hydraulic signals. The changes in the xylem tension are sensed by mechano-sensory channels directly, or through the lipid bilayer in which the channel resides. Complementary, slower (minutes to hours) transport of water-soluble signaling molecules (e.g. hormones), and sucrose translocation through the phloem (Shabala et al., 2016) can convey an important information about the external stress factors. However, there could be also another scenario and plant strategy to communicate different stimuli and induce the systemic responses to local stress. In some cases, a cross-talk is seen, in which one type of locally applied stress is capable of generating a protective response or acclimation to another type of biotic or abiotic stress (Mühlenbock et al., 2008; Szechyńska-Hebda et al., 2015, 2016a,b; Czarnocka et al., 2017). The environmental factors, like a sudden increase in light intensity, changes in temperature, limitation in water accessibility, or pathogen attack, all of them depress efficiency of CO_2_ assimilation due to reduction of stomatal conductance, but do not limit foliar absorption of light energy (Müller et al., 2001; Mullineaux and Karpiński, 2002; Holt et al., 2004; Baker, 2008). It results in the excess excitation energy (EEE) in photosystem II, then the changes in redox status of the photosynthetic electron carrier chain (namely the plastoquinone pool) and bursts of ROS, and finally photoinhibition and programmed cell death (PCD) (Mühlenbock et al., 2008; Wituszynska et al., 2013, 2015; Szechyńska-Hebda et al., 2015). EEE-induced cell death is regulated by the same genetic system as the hypersensitive response in disease resistance and the systemic acquired resistance (SAR) (Dangl and Jones, 2001; Mühlenbock et al., 2008). The information about locally induced EEE, unbalanced redox status of the cell, and microlesions is communicated to distant cells and organs via photoelectrophysiological signaling (PEPS), which consists of the electrical waves (with amplitude in range of 10-50 mV, and the nature corresponding to VP or SP), followed with the changes in nonphotochemical quenching, reactive oxygen species (ROS), expression of *ASCORBATE PEROXIDASE 2* (Szechyńska-Hebda et al., 2010) and the calcium (Ca^2+^) wave (Choi et al., 2017). PEPS can affect multiple physiological processes, e.g., respiration, transpiration, changes of ATP content, photosynthesis, transcription and translation of specific proteins, the synthesis of hormones such as ethylene, salicylic acid and jasmonic acid, in the tissues distant from those, which perceives the stimulus (Mühlenbock et al., 2008; Karpiński et al., 2013; Szechyńska-Hebda and Karpiński, 2013; Gilroy et al., 2016). Finally, the adjustment of such signaling components leads to induction of the systemic acquired acclimation (SAA) to abiotic stresses and simultaneously SAR to pathogens (Karpiński and Szechyńska-Hebda, 2010; Baluška, 2013; Karpiński et al., 2013; Figure 1).

The plant cells are able to physiologically “memorize” episodes of the EEE and following PEPS, and use this “memorized” information to improve their chances of survival in the future. It was demonstrated that fast development of infection has occurred in *Arabidopsis* plants grown under LL conditions. When the plants were infected prior to the excess light treatment, the bacteria initiated the wide-spread infection process too. However, in plants infected 1, 8 or 24 h after the excess of white or red light incident, bacteria growth was effectively inhibited. A partial exposition of *Arabidopsis* rosette to excess light has also induced the acclimation and defense responses in systemic tissues (Szechyńska-Hebda et al., 2010). Thus, the long-distance systemic signaling of EEE incident has two consequences. Coordination of electrical signals and wave-like changes in NPQ, H_2_O_2_, *APX1, APX2*, and Ca^2+^ allows the whole plant to prepare for future challenges, like abiotic and biotic stresses (Figure 1). However, one should hypothesize, that changes in the components accompanying the electrical signal allow to differentiate the type of response.

To enable short and long distance electrical signaling to induce SAA and SAR responses, different compartments of the cell and then different cells must be interconnected. At cellular level, chloroplasts can form a cellular network of extended chloroplast envelope membranes, known as stromules, to connect each other, and various organelles (e.g., the nucleus and plasma membrane). Some evidences suggest that this network of stromules is important for signaling and induction of acquired acclimation and resistance. The stromules are formed in response to light-sensitive redox signals within the chloroplast. Their number increases during the day; after treatment with specific inhibitors of the photosynthetic electron transport (DCMU, 3-(3,4-dichlorophenyl)-1,1-dime-thylurea, DBMIB, 2,5-dibromo-6-isopropyl-3-methyl-1,4-benzoquinone); and as an effect of ROS production, specifically in the chloroplast (Brunkard et al., 2015). Similarly, chloroplasts form stromules during infection or exogenous application of hydrogen peroxide (H_2_O_2_) and salicylic acid (SA). Numerous stromules have surrounded nuclei during defense response and these connections correlated with an accumulation of chloroplast-localized NRIP1 defense protein and H_2_O_2_ in the nucleus (Caplan et al., 2015). At intercellular level, AP and VP can propagate through plasmodesmata of the bundle sheath cell layer (Szechyńska-Hebda et al., 2010; Sager and Lee, 2014). The electrochemical potential propagation is effective, provided there is the membranes integrity (plasma membrane and their extension, i.e. the plasmodesmata—plasma membrane of another cell). Indeed, plasmodesmata are highly dynamic intercellular channels in the cell wall, containing, at its axial center the strands of the endoplasmic reticulum that are continuous between cells (Burch-Smith and Zambryski, 2010). Another path of the electrical signal propagation from cell to cell, independent of the PD, is depolarization of the cell plasmamembrane of an adjacent cell without direct connection (Gilroy et al., 2016). In both cases, resistance is too high for electrical waves to travel over distances larger than few neighboring cells. Therefore, other components could be involved, to enable more active electrical signaling, and it is hypothesized, that changes in the photosynthetic electron transport in light-treated chloroplasts are required to electrical signal propagation. If mechanical damage, treatment with LaCl_3_ (inhibitor of ion channels activity) or DCMU were made to the central vein in petiole of a leaf directly exposed to excess light, then such a leaf was not able to communicate SAA by electrochemical signaling. As a consequence, the expression of SAA marker genes (*APX1* and *APX2*) was not changed, and further SAA and SAR were not induced (Mühlenbock et al., 2008; Szechyńska-Hebda et al., 2010).

The chloroplasts in bundle sheath cells of veins have a minor role in the photosynthetic yield of leaves. Instead, they have a unique redox, hormonal and carbon metabolism (Kangasjärvi et al., 2009). Thus, a potential role for chloroplasts, in bundle sheath cell is to participate actively in autopropagation of electrochemical signaling. The relation between the chloroplasts and plasmodesmata-mediated intercellular signal propagation can be further supported by presence of the INCREASED SIZE EXCLUSION LIMIT 2 (ISE2) protein (Burch-Smith and Zambryski, 2010). Careful studies of ISE2-GFP localization reveal that ISE2 is a plastid protein acting as a regulation hub of both, plastid development and spatial organization of the plasmodesmata. The *ise1* and *ise2* mutation results in increased formation of twinned and branched plasmodesmata that facilitate the intercellular transport. Plasmodesmal permeability and remodeling can be also modified by cellular redox status and hydrogen peroxide presence, in both chloroplasts and mitochondria. Further, salicylic acid produced in chloroplasts, plays a role in closure of the plasmodesma through PLASMODESMATA-LOCATED PROTEIN 5 (PDLP5) (Lee et al., 2011) and in enhancing plasmodesmal complexity (Fitzgibbon et al., 2013). These data add plastid-to-nucleus signaling to plasmodesmata integrity as a critical factor in the plant communication network. Using plasmodesmata in conjunction with a phloem-based transport system, cells can deploy AP long-distance signaling between plant organs (Fromm and Lautner, 2007). Sieve tubes create a low-resistance pathway due to their plate pores and a continuum of plant plasma membranes. The length of a phloem vessel varies from several mm to several m, with diameters in the range from 1 to 100 mm. Ca^2+^ permeable channels located in the plasma membrane of the sieve tubes were shown to be associated with propagation of electrical signals induced by biotic and abiotic stresses (for review, see van Bel et al., 2014; Choi et al., 2017). Similarly, xylem participates in VP propagation and together with hydraulic waves it is responsible for systemic responses. Taken together, photosynthesis-dependent co-propagation of the electrical waves, ROS, calcium, and hydraulic waves, is a physiological prerequisite for induction of whole plant acclimation and systemic defense responses (SAA and SAR, Figure 1). SAA and SAR have been studied mainly in the leaves and flowering stems, however, the systemic propagation of signals is not limited to the aerial parts of plants. A role of the electrical signaling in roots was recently proposed. The electric field in the root rhizosphere reach up to 500 mV cm^−1^ and this is about two orders of magnitude higher than a threshold for electrotaxis for some soil-dwelling organisms. Thus, the leaf electric potential resulting from light or temperature fluctuations may propagate down to roots and modify the strength of electric fields in the rhizosphere, affecting the extent of root colonization by plant pathogens (Shabala et al., 2016). Due to the lack of chloroplasts in the root tissues, however, different physiological and molecular mechanism are involved in this signal propagation.

## Excess excitation energy and electrical signaling

Chloroplasts and photosynthesis seem to play a key role in triggering of electrical signals and adjustment of the components accompanying this signaling (PEPS). Plants, as sensible organisms, which cannot migrate when environmental conditions change and whose individual organs may experience different stress factors at the same time, need to take a benefit from specific mechanisms to cope with environmental heterogeneity. Many of the stress factors induce the excess excitation energy, over that required for optimal photosynthetic metabolism. In the classical view, the failure to dissipate EEE during stress is damaging to plants and often manifests as chlorosis, bleaching, or bronzing of leaves due to imbalanced metabolism of ROS. However, this could be the most universal system to use energy of the photons absorbed in the excess to improve survival chances of a whole plant (induction of the SAA and SAR). This mechanism has evolved in the photosynthetic organisms, since the light, in this context, is the most powerful and divergent factor. Quantity and quality of light in a plant canopy, changing day length, or spatial and temporal variations in the amount of radiation useful to photosynthesis, all of them affect plant growth and morphology, which in turn affect competition for light. Light was shown to modulate plant responses to almost all other stress factors (Karpiński et al., 2013). Thus, dynamic response of the plant to transient EEE incidents had an adaptive significance and determine plant Darwinian fitness.

Electrical signal propagation requires functional and photosynthetically active chloroplasts. The excess light triggers specific pattern of changes in plasma membrane electrical potential (Figure 1, Szechyńska-Hebda et al., 2010; Białasek et al., 2017). At the same time, local excess light exposure causes a rapid saturation of the photosynthetic reaction centers and their closure. This leads to a reduction in the fraction of energy utilized in photosynthesis, inhibition of the photosynthetic electron transport (photochemical reactions), the subsequent build-up of excess excitation energy in the photosynthetic membrane, and harmful ROS generation (Ruban, 2016). The mechanisms of photosynthetic electron transport and the electrical signaling, were proved to be interrelated. Excess light-triggered changes in plasma membrane potential, and then systemic propagation of the electrical potential were inhibited by DCMU, which prevents reduction of plastoquinone at photosystem II and generates singlet oxygen. The systemic propagation of the electrical signal was also deregulated by DBMIB, which prevents plastoquinol from reducing the cytochrome *b*_6_*f* complex, and generates superoxide (Szechyńska-Hebda et al., 2010; Ciszak et al., 2015; Gilroy et al., 2016; Białasek et al., 2017). Similarly, electrical signaling triggered by the excess light, had a reduced amplitude in *cad2* and *rax1* mutant (Szechyńska-Hebda et al., 2010). These mutants have deregulated glutathione synthesis, NPQ, the redox state of the PQ pool, *APX2* expression, SAA, and SAR (Ball et al., 2004). The second step of glutathione synthesis in the chloroplasts is important for regulation of *APX2* expression, light acclimatory and immune defense responses. Therefore, it is not surpassing, that EEE-triggered pattern of the electrical signal in the *apx2*-1 recessive mutant was also different, when compared to the wild type plant. Taken together, propagation of the electrical signal as well as other components of PEPS, are directly dependent on the excess energy in chloroplasts and efficiency of photochemical reactions (Szechyńska-Hebda et al., 2010).

**Figure 2:**
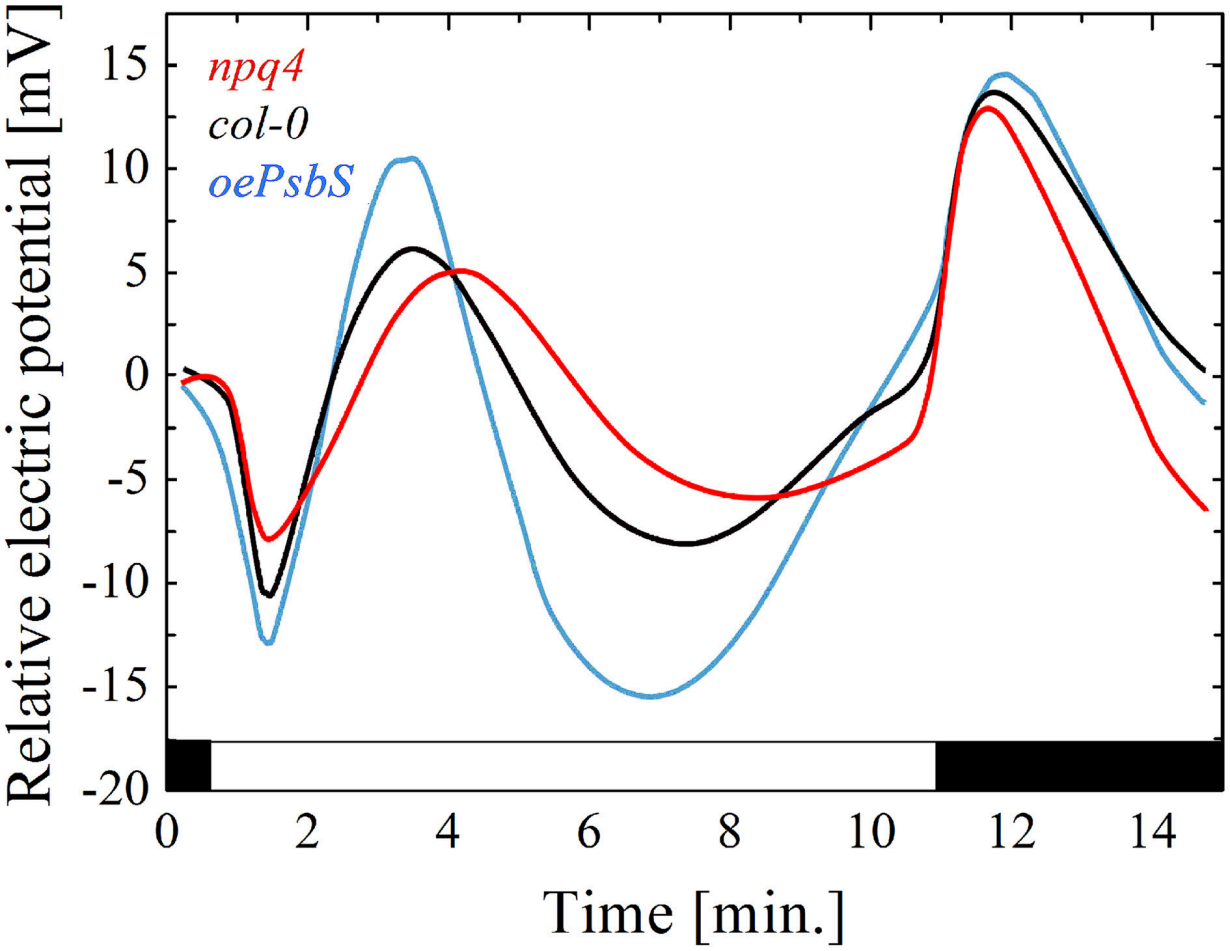
The electrical signals generated on the leaf surface in variable light conditions. Relative changes in the plasma membrane electrical potential were recorded for genotypes differing in PsbS protein content: red line, recessive mutant *npq4* devoid of PsbS; black line, WT ecotype Col*-*0; blue line, overexpressing line of *oePsbS*. Generally, the pattern of electrical signal detected on the leaf surface is reversed, when measured intracellularly. The recording by surface contact electrode detects a mixture of the signals from the three types of cells: guard, epidermal and mesophyll cells, and they can differ in their responses to light.

Considering above, all factors and mechanisms controlling the local level of the EEE in chloroplasts, other than photochemical reactions, can modify the electrical signaling and PEPS. Nonphotochemical quenching (NPQ). is a process in which light energy absorbed in excess, is dissipated into heat (Demmig-Adams et al., 2014; Ruban, 2016). It requires the trans-tylakoidal pH gradient and the proton sensor protein (PsbS) and prevents ROS generation. Some evidences indicate that NPQ, PsbS protein, H_2_O_2_ level, *APX2* are interrelated and play an important role in an electrical signal propagation inducing SAA and SAR. First, PsbS is required for SAA and light stress memory in *Arabidopsis (*Szechyńska-Hebda et al., 2010*;* Ciszak et al., 2015). The wild type rosettes exhibit a small reduction of fluorescence decay time in leaves directly exposed to excess light and in leaves undergoing SAA in low light conditions. However, recessive *Arabidopsis* mutant *npq4–1* has lost the ability to optimize florescence decay time after the excess light episode. It can be concluded that functional PsbS is required for optimization of the absorbed energy, thus quantum-molecular properties of PSII complexes in local and systemic leaves undergoing SAA. Second, PsbS is essential for zeaxanthin-dependent conformational changes in the thylakoid membrane that in turn are necessary for ΔpH-dependent regulation of NPQ (qZ). The lack of PsbS protein and dysfunction of the NPQ process influence the spatial and temporal pattern of the electrical membrane potential (Figure 2). Mutant *npq4* had small amplitude of plasma membrane potential after the light switching on and off, whereas plant with the PsbS overexpression (*oePsbS*) had an increased amplitude of membrane potential in comparison to the wild type plants. The kinetics of the electrical response was also different. The hyperpolarization, depolarization and subsequent repolarization of the plasma membrane of *npq4* mutant occurred within 9 min after switching on the light, while within seven and 6 min for wild type and *oePsbS*, respectively. Although, SAA and SAR are triggered by integrated wavy-like changes in the electrical signal, NPQ, H_2_O_2_, and *APX2*, the role of NPQ in the alternation of a membrane potential seems to be principal. Despite the higher *APX2* transcript level has been detected in *npq4* leaves (Szechyńska-Hebda et al., 2010), it did not prevent such considerable changes in electrical potential of its plasma membrane (Figure 2). Third, the photoelectrophysiological signaling, wavy-like changes in NPQ and H_2_O_2_, redox status of the glutathione and PQ pools, hormonal circuits (salicylic acid, jasmonic acid, ethylene), and the cellular light “memory” (Mühlenbock et al., 2008; Szechyńska-Hebda et al., 2010; Karpiński et al., 2013) were proposed as the mechanisms coordinating light acclimation (SAA), immune defenses (SAR), photosynthesis, transpiration, and developmental processes in the plants. The optimization of these processes are performed in a way similar to that defined by the algorithm of the cellular automation (Peak et al., 2004). When, leaves have experienced the patchy changes in *F*_v_/*F*_m_, NPQ, stomatal aperture, they “calculated” such changes to optimize cellular metabolism in leaves exposed to light and in leaves undergoing SAA.

The electrical signal propagation after the excess light treatment, mechanical wounding, burning, and current stimulation can also inactivate photosynthesis in systemic untreated tissues (Szechyńska-Hebda et al., 2010; Sukhov et al., 2015; Sukhov, 2016). The decrease of the effective quantum yields of photosystem I and II, reduction of CO_2_ assimilation rate, increase of NPQ were observed in the first 10–20 min after stimulation (Szechyńska-Hebda et al., 2010; Sherstneva et al., 2015; Sukhov et al., 2015; Sukhov, 2016). The photosynthetic responses were absent, if the electrical signals (PEPS) triggered by EEE do not propagate to systemic tissues or propagate with strongly reduced amplitudes (Szechyńska-Hebda et al., 2010), and it shares similarities with other types of electrical signals and stimuli (Sukhov et al., 2015). The strongest response was observed in the inter-vein area, where the photosynthetically active mesophyll cells are located. However, the photosynthetic changes in the veins were more rapid, thus suggesting the spread of electrical signals *via* the veins into the mesophyll cell (Koziolek et al., 2004; Białasek et al., 2017). Considering systemic changes in photosynthesis, the AP is less effective than VP. It can result from the restriction of AP spread in phloem without reaching the mesophyll cells, or from the stimulation of stomata opening. In most cases, the systemic photosynthetic response depends linearly on the VP amplitude (Sherstneva et al., 2015), thus the distance from site of stimulus. However, VP have ability to spread even over dead tissues zones and is not restricted in its propagation. Furthermore, VP induces stomata closure (via membrane depolarization, increased Ca^2+^ concentration, induction of ABA and JA), negatively influencing photosynthesis (Pavlovic, 2012). Electrical signals arising at the plasma membrane change ionic status of the cell (Figure 1). Probable mechanisms of photosynthesis inactivation involve H^+^ and (or) Ca^2+^ influxes. The reversible inactivation of the plasma membrane H^+^-ATPase during the electrical signals propagation (mainly VP) has an important consequence, i.e., acidification of the cytoplasm and alkalization of the apoplast of plant cells, the extend of which correlates with the VP amplitude (Sherstneva et al., 2015). Electrical signals transmitted to the thylakoid membranes also influence pH gradient at the thylakoid membrane, photoelectrochemical field, the charge separation, recombination reactions in PSII (Koziolek et al., 2004; Bulychev and Kamzolkina, 2006), and energy-dependent NPQ (ΔpH-dependent qE) (Krupenina and Bulychev, 2007). However, changes in PSII including an increase in NPQ may be determined by intensity of the stimuli and thus the parameters of VPs (Vodeneev et al., 2017). Hypothetical mechanism consists of the increase in cytoplasmic Ca^2+^ level via opening of plasma membrane Ca^2+^ channels and the release of Ca^2+^ from intracellular stores. Since, the chloroplast envelope is endowed with a light-dependent Ca^2+^ uniport, the accumulation of Ca^2+^ in the stroma is ensured. Candidates for the transport across the chloroplast envelope are proteins: ACA1, HMA1, GLR3.4, MSL2/3, and PPF1. The predominant portion of the chloroplastic Ca^2+^ can be bound to the negatively charged thylakoid membranes or to calcium-binding proteins, and thus Ca^2+^ influx can be linked to photosynthetic electron transport via the membrane potential. Further, the import of Ca^2+^ across the thylakoid membrane has been shown to be dependent on a light- or ATP-induced transthylakoid proton. Excess light-induced acidification leads also to reversible release of Ca^2+^ from PSII and an inactivation of oxygen evolution gradient (detailed review by Hochmal et al., 2015). The further development of the response can be associated with a decrease in the activity of the dark reactions of photosynthesis (Sherstneva et al., 2015). Accumulation of Ca^2+^ in the chloroplast suppresses CO_2_ fixation (Pottosin and Shabala, 2016), and K^+^ transport is important to these processes. In *Arabidopsis*, TPK3, a member of a tandem-pore K^+^ channel family, is localized to the thylakoids. Plants silenced for the *TPK3* gene showed lower CO_2_ fixation and altered NPQ (Carraretto et al., 2013). Modulation of this channel by natural factors was unexplored, although TPK3 appeared to display a higher activity at high (>100 mM) Ca^2+^ (Pottosin and Shabala, 2016). Calcium is also believed to be important for the carbon metabolism by the regulation of several key enzymes, including fructose 1,6-bisphosphatase and sedoheptulose 1,7-bisphosphatase of the reductive pentose phosphate cycle (Hochmal et al., 2015). VP-induced stomata closure increases the ${\text{HCO}}_{3}^{-}$: CO_2_ ratio and decreases CO_2_ net uptake into chloroplasts (Pavlovic et al., 2011; Gallé et al., 2013; Sukhov et al., 2016). An increase in the external Ca^2+^ concentration has been shown to be crucial for regulating the stomatal aperture in *A. thaliana*. Usually, the closure of stomata *via* an increase of the external Ca^2+^ concentration is accompanied by transient and repetitive elevations in [Ca^2+^]_*cyt*_. Calcium sensor protein CAS is required for proper stomatal regulation in response to elevations of external Ca^2+^ through the modulation of cytoplasmic Ca^2+^ dynamics (Hochmal et al., 2015). Carbonic anhydrases can mediate the changes in ${\text{HCO}}_{3}^{-}$: CO_2_ ratio in light-dependent manner (Dąbrowska-Bronk et al., 2016). The decrease in the CO_2_ flow can also be linked to the changes in abscisic acid accumulation observed during VP generation (Figure 1, Sukhov et al., 2016). The decrease in CO_2_ assimilation in response to VP was observed in different plants with different extend: 22% of the assimilation under illumination in pumpkin, 55% decrease in the assimilation in pea, decreased by ~100% of the CO_2_ absorption under illumination in geranium (Sherstneva et al., 2015). Furthermore, cyclic electron flow could be partially rescued by an increase in the extracellular Ca^2+^ concentration and CAS. Cyclic electron flow and qE are interconnected as cyclic electron flow participates in acidification of the thylakoid lumen, which is required for efficient qE. It also interconnects PSI and Ca^2+^-dependent control. Taken together these data underline primary role of calcium in regulation of the photo-protective mechanisms and chloroplast metabolism (Hochmal et al., 2015). It also suggests that photosynthesis and electrical signaling can be regulated/deregulated by the same or at least similar mechanisms in local and systemic tissues (Szechyńska-Hebda et al., 2010).

White, red and blue light stimulated similar temporal pattern of changes in the electrical membrane potential. However, amplitude and speed of the electrical signal can vary with quality of the light, and it suggests that apart from chloroplasts, different photoreceptors (phytochromes, cryptochromes, phototropins) can influence signals propagation in plasma membrane. Several photoreceptors can trigger cytosolic Ca^2+^ signals to stimulate changes in photosynthesis, e.g., phototropins in hypocotyls cells (Folta et al., 2003) and phytochromes in caulonema cells (Ermolayeva et al., 1997). Generally, VP and SP are preferably triggered by red light (Okazaki, 2002; Sukhov et al., 2016).

## Systemic regulation of excess light-responsive genes encoding ion channels

Approximately 5% of the *Arabidopsis* genome encodes integral membrane transport proteins, classified in 46 families containing ~880 members (Mäser et al., 2001). Some of the ion channels play a key role in the generation and sensing of electrical activity that depends on the light. They are involved in the exchange of the ions between extra-and intracellular space, across plasma membrane. Channels switch between transporting and non-transporting (open and closed) states by a stochastic process referred to as gating, is influenced by a variety of factors (e.g., voltage-, pH-, mechanically-gated) (Spalding, 2000). Many early studies have focused on the changes of membrane electrical potential and its local ionic mechanism during light to dark transition (Jeschke, 1976; Trebacz and Zawadzki, 1985; Trebacz et al., 1989). Although, the design of experiments can influence the pattern of electric signal (Figure 2, Białasek et al., 2017), in most cases, the dark (or low light) to light (or high light) transition results in a short hyperpolarization, followed by a depolarization, and repolarization of membrane (Szechyńska-Hebda et al., 2010). A proton pump, K^+^, Cl^−^, and Ca^2+^ ion channels were proposed to participate in the light-induced changes of a membrane potential (Figure 1, Dietrich et al., 2001; Živanović et al., 2005, 2015). The hyperpolarization can result from proton extrusion via the H^+^-ATPase, an electroenzyme residing in the plasma membrane at high density (Dietrich et al., 2001; Živanović et al., 2005, 2015) as well as from the activity of the voltage-dependent inward rectifying K^+^ channels (Yan et al., 2009). The mechanism of AP depolarization can occur *via* an influx of Ca^2+^ through voltage- or mechanically-gated Ca^2+^ channels. The elevated Ca^2+^ concentration triggers the sequence of events: activation of voltage-dependent anion channels and the efflux of Cl^−^, activation of the outward K^+^ channels and K^+^ efflux, gradual inactivation of Cl^−^ channels. The changes in activity of K^+^ and Cl^−^ channels lead finally to membrane repolarization and its return to level of resting potential. VPs and SPs are rather initiated through a transient inactivation of plasma membrane proton pump H^+^-ATPase, which causes a slow depolarization of the membrane potential (Fromm and Lautner, 2007). However, Ca^2+^, Cl^−^, and K^+^ channel activation might also participate in the process (Sukhov, 2016).

**Figure 3:**
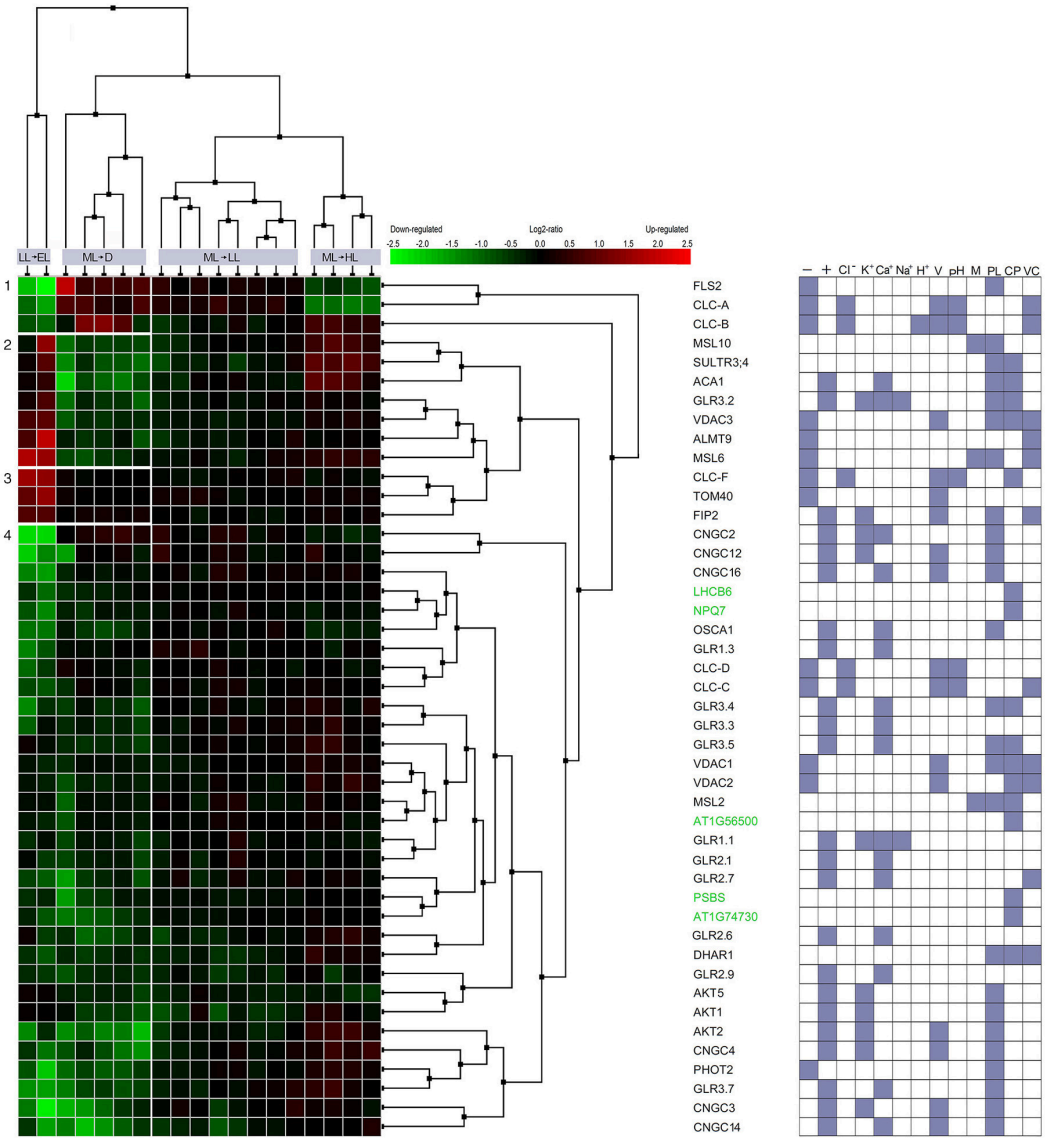
Co-expression of light-responsive genes involved in the flow of ions across the membranes and in the non-photochemical quenching. Multiple microarray and RNAseq experiments, as well as NCBI database were searched to select genes which respond to the excess light (induction of SAA and SAR) and are involved in ion transport across biological membranes in *Arabidopsis*. 273 genes with query “ion channel” and 10 genes for query “nonphotochemical quenching” were verified in the AmiGO 2 database. Finally, the Similarity Search Tool and Hierarchical Clustering of the Genevestigator were used to select 45 genes with the most closely correlated expression profiles during defined light conditions (Exp ID: AT-00467 and AT-00682). The transfer of the plant from low light (LL, 65 μmol photons m^−2^ s^−1^) to excess light (EL, 1300 μmol photons m^−2^ s^−1^), from moderate light (ML, 150 μmol photons m^−2^ s^−1^) to darkness (D), from ML to LL; and from ML to high light (HL, 400 μmol photons m^−2^ s^−1^) was compared. Left panel represents relative expression data of experimental vs control values. GO class was assigned to each gene according to NCBI and Gene Set Enrichment tool of Genevestigetor and the results is indicated in the right panel as: **-** anion channel activity; + cation channel activity; **Cl**^**−**^, **K**^**+**^, **Ca^2+^** **Na**^**+**^, **H**^**+**^, chloride, potassium, calcium, sodium and hydrogen ions channel activity; **V, pH, M** - voltage-, pH-, mechanically-gated ion channel activity; **PL, CP**, **VC**, protein localization in plasma membrane, chloroplast, vacuole.

It was suggested that a key component of the electrical waves kinetics and the tissue-specific nature of the spatial patterning of wave transmission may lie in the regulation of its channel activity rather than its gene expression pattern (Choi et al., 2016). However, the induction of vascular genes, i.e., *APX1, APX2, ZAT10*, can follow almost immediately electrical signal propagation. The exposure of older leaves on the rosette to very high light triggers the signal which moves within 15–30 min via the vasculature toward non-exposed younger rosette leaves (Figure 1), cauline leaves, and the stem of the floral bolt (Szechyńska-Hebda et al., 2010). The induction of EEE, SAA, and SAR marker genes have already been detected within 20–30 min at the same localization on the signal path (Karpiński et al., 1999; Rossel et al., 2007; Szechyńska-Hebda et al., 2010). Moreover, exposure to HL increased the expression of 360 genes and decreased the expression of 247 genes in distal leaves after 30 min (Rossel et al., 2007). In the same way, changes in expression of the genes encoding the ion channels or the proteins regulating ion channel activity take a part of light-dependent mechanism in the plant cells (Figure 3). The differentially expressed genes (in relation to untreated control plants) were detected 30 min after excess light exposure, and 60 min after shift to dark. Interestingly, significant changes in the expression were found, provided the light stress exceed defined threshold. The fluctuations in the range from low light to excess light and from moderate light to darkness have altered 20% of genes toward their induction and 80% of genes toward their suppression. Transfer of the plant from moderate light to low light or high light did not induce considerable changes in gene expression (Figure 3). It suggests analogy with depolarization, which needs to exceed a certain threshold to trigger AP, SAA and SAR (Karpiński et al., 1999; Szarek and Trebacz, 1999; Szechyńska-Hebda et al., 2010).

Genes encoding ion channels critical for the control of long-term responses during excess light fluctuations (that can trigger SAA and SAR) were clustered in several groups. Among the genes in cluster no 1, *FLS2* was down regulated during LL to EL shift and upregulated during ML to D shift. FLS2 is a plasma membrane receptor-like kinase (RLK), which can recognize a 22-aminoacid residue stretch of the flagellin protein from *Pseudomonas syringae*. FLS2 is involved in early signaling pathways such as: activation the anion channels (e.g., SLAH3) through cytosolic Ca^2+^ signals and inhibition the guard cell inward K^+^ channels. The regulation of these channels is important to rapid stomatal closure, thereby retarding pathogen invasion (Zhang et al., 2008; Guo et al., 2014; Deger et al., 2015), but also influencing photosynthesis. FLS2 complexes with BAK1 and BKK1 play a role in the regulation of *AtCLCs* expression (Guo et al., 2014). Indeed, genes encoding CLC-A, ion channel protein regulating the outward anion fluxes across the vacuolar membrane (Wege et al., 2014) and CLC-B, ion channel protein mediating ${\text{NO}}_{3}^{-}$/H^+^ exchange across the tonoplast (von der Fecht-Bartenbach et al., 2010), both were co-expressed with *FLS2* (Figure 3, Table 1). They suppression under EL treatment suggests that SAR (and SAA) induced by EL (Karpiński et al., 1999) would not overlap with FLS2- and CLCs-dependent pathways and resistance. Similarly, genes encoding AtCLC-C and AtCLC-D, the anion transporters negatively regulating pathogen-associated molecular pattern (PAMP)-triggered immunity (PTI) and stomatal movement (Jossier et al., 2010; Guo et al., 2014), both were down-regulated during fluctuating light conditions (cluster of the genes no 7). In contrast, *CLC-F*, a chloride channel protein, was upregulated during shift to EL (cluster of the genes no 3), but its involvement in light-triggered responses could be related to cellular localization in the chloroplast and Golgi apparatus. Thus, *CLC-F* is rather involved in the second mechanism described below.

**Table 1:** The genes involved in the flow of ions across the membranes and in the non-photochemical quenching, which expression is graphically presented on Figure 3.

	**Gene/product**	**Gene/product name**	
1	FLS2	AT5G46330	Leucine-rich receptor-like protein kinase family protein
	CLC-A	AT5G40890	Chloride channel A
	CLC-B	AT3G27170	Chloride channel B
2	MSL10	AT5G12080	Mechanosensitive channel of small conductance-like 10
	SULTR3;4, MSL1	AT3G15990	Sulfate transporter 3;4
	ACA1	AT1G27770	Autoinhibited Ca2+-ATPase 1
	GLR3.2, GLUR2	AT4G35290	Glutamate receptor 2
	VDAC3	AT5G15090	Voltage dependent anion channel 3
	ALMT9	AT3G18440	Aluminum activated malate transporter 9
	MSL6	AT1G78610	Mechanosensitive channel of small conductance-like 6
3	CLC-F	AT1G55620	Chloride channel F
	TOM40	AT3G20000	Translocase of the outer mitochondrial membrane 40
	FIP2	AT5G55000	Potassium channel tetramerization domain-containing protein
4	CNGC2, DND1	AT5G15410	Cyclic nucleotide-regulated ion channel family protein
	CNGC12	AT2G46450	Cyclic nucleotide-gated channel 12
	CNGC16	AT3G48010	Cyclic nucleotide-gated channel 16
	LHCB6	AT1G15820	Light harvesting complex photosystem II subunit 6
	NPQ7	AT1G65420	Antigen receptor-like protein
	OSCA1	AT4G04340	ERD (early-responsive to dehydration stress) family protein
	GLR1.3	AT5G48410	Glutamate receptor 1.3
	CLC-D	AT5G26240	Chloride channel D
	CLC-C	AT5G49890	Chloride channel C
	GLR3.4	AT1G05200	Glutamate receptor 3.4
	GLR3.3	AT1G42540	Glutamate receptor 3.3
	GLR3.5	AT2G32390	Glutamate receptor 3.5
	VDAC1	AT3G01280	Voltage dependent anion channel 1
	VDAC2	AT5G67500	Voltage dependent anion channel 2
	MSL2	AT5G10490	MSCS-like 2
	SOQ1	AT1G56500	Suppressor of quenching 1
	GLR1.1	AT3G04110	Glutamate receptor 1.1
	GLR2.1	AT5G27100	Glutamate receptor 2.1
	GLR2.7	AT2G29120	Glutamate receptor 2.7
	PsbS, NPQ4	AT1G44575	Chlorophyll A-B binding family protein, Nonphotochemical quenching 4
	RIQ2	AT1G74730	Transmembrane protein, putative
	GLR2.6	AT5G11180	Glutamate receptor 2.6
	DHAR1	AT1G19570	Dehydroascorbate reductase
	GLR2.9	AT2G29100	Glutamate receptor 2.9
	AKT5	AT4G32500	K+ transporter 5
	AKT1	AT2G26650	K+ transporter 1
	AKT2	AT4G22200	Potassium transport 2/3
	CNGC4	AT5G54250	Cyclic nucleotide-gated cation channel 4
	PHOT2	AT5G58140	Phototropin 2
	GLR3.7/GLR5	AT2G32400	Glutamate receptor 5
	CNGC3	AT2G46430	Cyclic nucleotide gated channel 3
	CNGC14	AT2G24610	Cyclic nucleotide-gated channel 14

Up-regulated genes during plant shift to EL, and down-regulated genes during plant transfer to dark, were clustered into group no 2. Among them, genes encoding MSL10 and two other members of the MSL family (MSL1, MSL6), were found. They are mechanically-gated ion channels, responsible for anion transport, functioning as osmotic safety valves and releasing osmolytes under increased membrane tension. MSL10 is located in plasma membrane and regulates PCD signaling. High-level expression of *MSL10*-GFP in *Arabidopsis* induced small stature, H_2_O_2_ accumulation, ROS- and CD-associated gene expression (Maksaev and Haswell, 2012; Veley et al., 2014). A role of MSL1 (SULTR3;4) is diverse. MSL1 participate in sulfate and Pi translocation (Cao et al., 2013), and along with MSL2 and MSL3, controls plastid division and organelle morphology (Haswell et al., 2008; Wilson et al., 2011). MSL6 is located in plasma membrane and plasmodesma, what suggests its role in transportation or signal propagation. Further, two genes involved in calcium transport were found in the cluster no 2. ACA1, a calmodulin-activated calcium channel protein, is the most promising candidate for the calcium import across the inner chloroplast envelope membrane (Carraretto et al., 2016). GLR3.2 (AtGluR2), an intracellular ligand-gated ion channel protein, maintains cellular calcium, potassium, and sodium ion homeostasis. Its deregulation led to Ca^2+^ deficiency, hypersensitivity to Na^+^ and K^+^ ionic stresses, death of the shoot apex, necrosis and deformation of leaves. The promoter of the *AtGluR2* gene was active in vascular tissues, particularly in cells adjacent to the conducting vessels (Kim et al., 2001). Finally, two genes encoding proteins with anion channel activity were upregulated in EL and downregulated in dark. VDAC3, anion channel being the most abundant in plasma membrane (Robert et al., 2012) is involved in the hypersensitive response, regulation of seed germination, and response to cold; whereas ALMT9, is a malate-activated vacuolar chloride channel, having a major role in controlling stomata aperture (De Angeli et al., 2013). This set of genes can be extended with three additional genes encoding proteins with poorly known physiological function. They are clustered into the group no 3, as induced in response to shift from LL to EL, but with the expression unchanged in dark. First, CLC-F, a voltage-gated chloride channel protein, being a component of the outer envelope membrane of chloroplasts (Teardo et al., 2005) or Golgi membranes (Marmagne et al., 2007). Consistent with the plastidial localization of CLC-F, the protein is expressed in leaf etioplasts and chloroplasts, but not in root tissue (Teardo et al., 2005). CLC-F may operate at contact sites, where it may sense voltage and regulate chloride flux into the stroma. Second, TOM40 is exclusively localized to outer mitochondrial membrane and is mainly involved in pore formation, protein targeting and import into mitochondria. However, voltage-gated anion channel activity and ion transport were also considered (Werhahn et al., 2001; Lister et al., 2004). Third, FIP2 is voltage-gated potassium channel located in plasma membrane and vacuole.

Considering localization and function of the above proteins that are induced in EL, but suppressed or unchanged in dark, the tight relations between chloroplasts and plasma membrane, between photosynthesis and long-distance electrical signals propagation, and between SAA and SAR can be assumed (Figures 1, 3). Although, the cell is compartmented, there is no doubt that ionic changes in one organelle can influence the others; however, studies clarifying these interrelations are rare. Indirect evidences suggest possible mechanisms. The one of the first response is pH change in both, the cytosol and chloroplast. Hyperpolarization of plasma membrane, induced during EL, can be mediated by H^+^-ATPase activity. H^+^-ATPase was shown to play a main role in VP and SP generation, and participate in AP development (Sukhov, 2016). In chloroplast, EL leads to the generation of a proton motive force (pmf), which comprises a proton gradient (ΔpH) and a transmembrane electrical potential difference (ΔΨ). The changes in pH induce fluxes of Cl^−^, K^+^, and Ca^2+^ through plasma membrane and chloroplast envelope (Figure 1). Ca^2+^ dependent Cl channel activation during AP and VP was suggested as the potential mechanism of pH changes in cytosol; and a light-induced anion accumulation in thylakoids, including Cl^−^ concentration on the luminal side, was also recorded during shift of the plant to light conditions. Electrical signal-dependent K^+^ efflux across plasma membrane could influence the pH, because fixed negative charges can concurrently bind H^+^ and K^+^. Similarly, some potassium ion channels of the thylakoid membrane, can modulate the composition of the pmf through ion counterbalancing (e.g., Ca^2+^ and H^+^) (Carraretto et al., 2013). Potential-dependent Ca^2+^ channels activate AP; and ligand-dependent and mechanosensitive Ca^+^ channel trigger VP. Then, Ca^2+^ uptake by energized thylakoids can occur via Ca^2+^/H^+^ antiport mechanism. These mechanisms are known to inactivate the Calvin–Benson cycle (Figure 1, Lautner et al., 2005; Fromm et al., 2013; Carraretto et al., 2016), by inducing fast and long-term inactivation of photosynthesis (for detailed review refer Sukhov et al., 2016). Altogether, one has to assume that the activity of ion channels localized in chloroplast and plasma membrane is synchronized and the ion channels influence each other as a feedback.

Systemic changes in gene expression (including those genes that are involved in ion channel activity) are an outcome of the electrical signals and transduction of accompanying signaling components. However, the gene clustering (Figure 3, Table 1) indicated clearly, that only part of ion channels is important for mechanisms described above. The upregulation of the genes from cluster 2 indicates them as potential functional candidates and/or markers of long term acclimation (SAA) and defense (SAR). The other genes encoding ion channels are down-regulated (Figure 3, Table 1, cluster no 4). They are co-expressed with five genes involved in NPQ responses, i.e., *LHCB6, NPQ7, SOQ1, PsbS, RIQ2* (Table 1, genes marked in green). Without doubt, the electrical signaling is costly. Therefore, probably the most likely explanation is, that in order to establish a favorable energy balance, upregulation of genes directly related to systemic response induction, SAA and SAR, need to be compensated by the down-regulation of genes involved in signaling (effect post-factum).

On a final note, it should be pointed out that unicellular organisms, plants and animals face many of the same problems. The most basic problem to solve is ensuring the energy to life, reproduction, acclimation and defense. To increase the chances of survival and proliferation, organisms at each level of organization need to communicate internal and external factors to adjust energy status in fluctuating environment. The electrical signaling may have evolved in many different ways, however, at the most basic level, the spread of electrical signals is similar and serves as the most universal system for intracellular, intercellular, organ-to-organ and organism-to-organism communication. For plants, solar energy is the basis of functioning. Therefore, integration of electrical signals with additional components that are dependent on energy absorption provides a powerful mechanism that holistically control homeostasis of the plant. Using network of the hyphae of mycorrhizal fungi, plants can even collectively manage absorbed energy and recourses, help to survive each other, and regulate homeostasis of the plant community. Therefore, science fiction world presented in James Cameron's Avatar movie seems there is quite close to the real sciences, particularly plant neurobiology.

## Author contributions

The authors made equal intellectual contributions to the work and approved it for publication.

### Conflict of interest statement

The authors declare that the research was conducted in the absence of any commercial or financial relationships that could be construed as a potential conflict of interest.
